# How Immunosenescence and Inflammaging May Contribute to Hyperinflammatory Syndrome in COVID-19

**DOI:** 10.3390/ijms222212539

**Published:** 2021-11-21

**Authors:** Ludmila Müller, Svetlana Di Benedetto

**Affiliations:** Max Planck Institute for Human Development, Lentzeallee 94, 14195 Berlin, Germany; dibenedettos@ymail.com

**Keywords:** aging, immunosenescence, inflammaging, cytokine storm, senescence-associated secretory phenotype (SASP), CMV, hyperinflammatory syndrome, COVID-19

## Abstract

Aging is characterized by the dynamic remodeling of the immune system designated “immunosenescence,” and is associated with altered hematopoiesis, thymic involution, and lifelong immune stimulation by multitudinous chronic stressors, including the cytomegalovirus (CMV). Such alterations may contribute to a lowered proportion of naïve T-cells and to reduced diversity of the T-cell repertoire. In the peripheral circulation, a shift occurs towards accumulations of T and B-cell populations with memory phenotypes, and to accumulation of putatively senescent and exhausted immune cells. The aging-related accumulations of functionally exhausted memory T lymphocytes, commonly secreting pro-inflammatory cytokines, together with mediators and factors of the innate immune system, are considered to contribute to the low-grade inflammation (inflammaging) often observed in elderly people. These senescent immune cells not only secrete inflammatory mediators, but are also able to negatively modulate their environments. In this review, we give a short summary of the ways that immunosenescence, inflammaging, and CMV infection may cause insufficient immune responses, contribute to the establishment of the hyperinflammatory syndrome and impact the severity of the coronavirus disease 2019 (COVID-19) in elderly people.

## 1. Introduction

**Figure 1:**
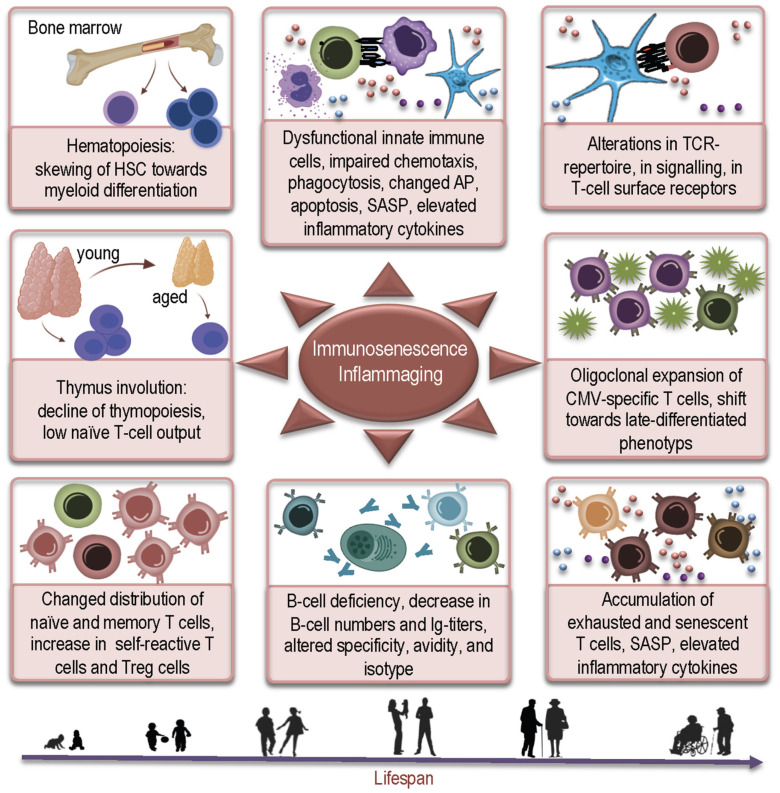
Schematic illustration of factors contributing to immunosenescence and inflammaging. HSC: hematopoietic stem cell; AP: antigen presentation; SASP: senescence associated secretory phenotype; TCR: T-cell receptor; CMV: cytomegalovirus; Treg: regulatory T-cell.

Changes in the human immune system accompanying aging represent a universal, complex and multidimensional process, the spectrum of which is generally referred to as immunosenescence. This phenomenon of age-related dynamical remodeling of the immune system is now considered as a process of physiological adaptation to the aged microenvironment [1,2]. Many factors and mechanisms are attributed to immunosenescence (Figure 1), including defects in hematopoiesis; thymus involution; and changes in the formation, maturation, migration, and homeostasis of peripheral lymphocytes [2]. Aging affects all levels of both innate and adaptive arms of the immune system and is commonly accompanied by an increased tendency for low-grade inflammation thought to be a contributing or even causative factor for a multitude of clinical conditions in elderly people [3,4].

Functional impairments of the immune system with aging are at least partly related to the age-related dysregulation of hematopoiesis [5]. Some of the central mechanisms contributing to the deterioration of the immune competence are changes in the lymphoid and myeloid lineage during hematopoiesis, resulting in skewing towards myeloid differentiation. Another major event that is thought to have a pronounced influence on the aging immune system is the process of gradual thymic involution, which begins at puberty and continues throughout life [6]. This is a conserved developmental event but contributes to immunosenescence in later life by decreasing the capacity to generate new naïve T-cells.

The availability of naïve T lymphocytes is essential for the development of adaptive immunity against new challenges. Thus, age-related changes in hematopoiesis combined with thymic involution contribute at least partly to the diminished immune functions of the cells of the innate and the adaptive immune system [7].

Innate immune cells possess the exclusive capacity to respond immediately to pathogens in a generic way by activating such defense mechanisms as phagocytosis; inflammatory reactions; activation of the complement system; and recruitment of essential cells—such as eosinophils, neutrophils, macrophages, natural killer cells (NKs), and dendritic cells (DCs)—to sites of detected infection. Various age-associated functional impairments have been reported in the phagocytic mechanisms of these cells, chemotaxis, the generation of toxic free radicals, and the susceptibility to apoptosis [8]. Some of the diminished functions in neutrophils of advanced age were found to be associated with altered production of chemokines and cytokines; reduced expression levels of receptors recognizing pathogen-associated molecular patterns (PAMPs)—such as Toll-like receptors (TLR); and lower-level expression of the major histocompatibility complex class II (MHC-II) molecules [9]. The phagocytic and functional features of macrophages are also impaired with age, accompanied by altered production of reactive oxygen species, such as NO_2_ and H_2_O_2_, and pro-inflammatory cytokines [10,11,12].

A disruption in the fine balance between adaptive and innate immunity may lead to dysregulation of the effector cells and their mediators, causing inflammatory conditions. Immunosenescence is characterized by not only innate-cell-induced chronic sterile inflammation, but also by adaptive-immune-cell-induced basal inflammation associated with T-cell immunosenescence. The latter is manifested by restricted diversity of the T-cell receptor (TCR) repertoire, the accumulation of senescent and exhausted memory T-cells with functional impairments, more self-reactive T-cells, and more functionally enhanced polyclonal regulatory T (Treg) cells [13,14].

A persistent infection with cytomegalovirus may have a strong modulatory effect on the immune system, leading to a loss of the T-cell diversity, functional alterations, and eventually immunosenescence. Lifelong infection with CMV results in the expansion and accumulation of late-stage differentiated effector memory T-cells in the peripheral circulation [15]. In people infected with CMV, the elderly especially, a large proportion of circulating CD8^+^ T-cells are specific for CMV [16]. This phenomenon of sequential increases in CMV-specific CD8^+^ T-cells over the lifespan is known as “memory inflation” [17,18] and was suggested to be responsible for high mortality [15,19,20,21,22] in the elderly and with frailty and impaired survival [23,24,25].

Adaptive and innate immunity are involved in extremely regulated and highly specific multidirectional interactions between innate and adaptive cells—such as T and B lymphocytes. Therefore, it is not surprising that the age-related alterations in this balance affect all levels of the immune response. Low numbers of functional immunoglobulin-secreting B-cells in elderly people have been found consistently, correlating with decreased titers of antigen-specific antibodies. Not only B-cell subsets themselves, but also the repertoire of immunoglobulins produced by them, are altered—in both specificity and isotype. The consequence of these changes may well be the shorter duration of the humoral response—due to the reduced B-cell capacity for providing specific primary and secondary responses—observed in elderly individuals [26,27]. Age-associated deficiencies in T helper cells can have negative modulatory effects on their cognate interactions with B-cells by reducing the quality of antibodies in terms of their avidities, and their titers [28].

The B-cell deficiency together with the TCR-repertoire shrinkage may lead to, or at least contribute to, the lowered responses to new pathogens and to the generally poor vaccination responses in this age group [15]; but until recently, surprisingly, an insufficient amount of data were available on this issue. The world-wide pandemic caused by severe acute respiratory syndrome coronavirus 2 (SARS-CoV-2) dramatically changed this situation, as age-related heterogenicity in the immune system, along with the differences in the immune and inflammatory responses, may have a decisive effect on and determine the spectrum of COVID-19 severity [29].

## 2. How Aged Bone Marrow and Thymic Aging May Contribute to Susceptibility to COVID-19

### 2.1. Age-Related Changes in Hematopoiesis and COVID-19

**Figure 2:**
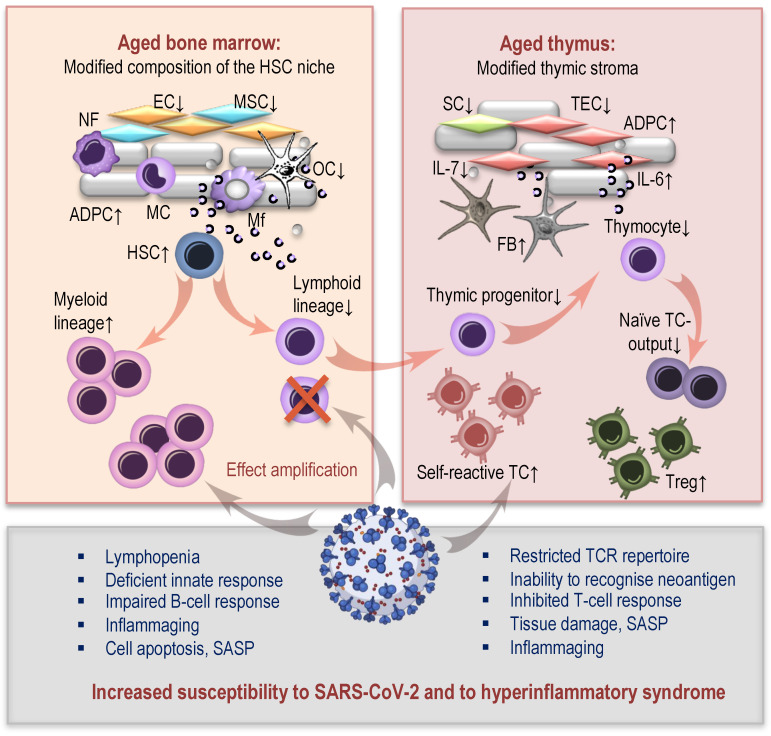
Age-related changes in hematopoiesis and thymopoiesis and their impacts on SARS-CoV-2 infection. NF: neutrophile; EC: endothelial cell; MSC: mesenchymal stromal cell; OC: osteoclast; ADPC: adipocyte; Mf: macrophage; MC: monocyte; HSC: hematopoietic stem cell; SC: stromal cell; TEC: thymic epithelial cell; IL: interleukin; TCR: T-cell receptor; Treg: regulatory T-cell; SASP: senescence-associated secretory phenotype; TC: T-cell.

Age-related changes in hematopoiesis, which play a pivotal role in the continuous processes of the renewal of blood and immune cells, may at least in part be responsible for the functional impairments of the immune system and influence the fate of a COVID-19 patient (Figure 2, left). Throughout life, adult hematopoietic stem cells (HSCs), which consist of a heterogeneous subgroup of multipotent and unipotent progenitors, produce the entire cell spectrum of lymphoid and myeloid lineages of the immune system [30,31]. The reciprocal communications between different cells of the HSC microenvironment (consisting of endothelial and mesenchymal stromal cells, adipocytes, macrophages, neutrophils, osteoclasts, and regulatory T-cells) appear to be disturbed in the aging bone marrow. The hematopoietic compartment gradually shrinks and is progressively replaced by fatty tissue [32,33,34].

The consequences of these age-related changes in the HSC niche may include: insufficient DNA-repair capacity and epigenetic changes; increases in HSC numbers with a simultaneous decrease in their homing efficiency; myeloid skewing of their differentiation potential, leading to downregulation of lymphoid differentiation genes and upregulation of myeloid differentiation genes. An aging HSC is characterized by an accumulation of DNA damage and telomere shortening, combined with an increase in intracellular reactive oxygen species—the events that eventually lead to its reduced functionality and genomic instability [35,36,37,38,39]. Age-associated skewing towards myeloid differentiation may represent one of the decisive mechanisms contributing to the deterioration of the immune system’s competence in elderly individuals [37,40,41], which can impact susceptibility to SARS-CoV-2 infection.

The results of a recent study [5] have demonstrated dysregulated hematopoiesis in patients with severe COVID-19 compared to a control group. This manifested in a significant accumulation of immature myeloid progenitors, a dramatic reduction of lymphoid progenitors, and upregulation of transcription factors which are important for HSCs and for their downstream progenitor differentiation. Thus, we can speculate that COVID-19 appears to have similar effects on lymphopoiesis as aging itself, and an infection with SARS-CoV-2 may possibly be able to amplify these effects. However, more studies are needed to understand these compound interactions.

### 2.2. Thymic Aging and COVID-19

Age-related thymic involution (Figure 2, right) is characterized by a progressive reduction in the thymic epithelial areas engaged in active thymopoiesis [42] with a concomitant increase in the perivascular areas comprising connective, adipose, and fibrous tissues [33]. This process is accompanied by a changed immunometabolism in the thymic microenvironment [43], reduced production of thymo-supportive interleukin (IL)-7 [44] and enhanced secretion of thymo-suppressive mediators. These modifications adversely impact the functions and number of thymic epithelial and stromal cells, which has negative consequences for thymopoiesis. The age-related decline in the thymic function reduces naïve T-cell output and leads to a contraction of the T-cell repertoire’s diversity, thereby reducing the ability to recognize neo-antigens, resulting in increased infection susceptibility [6,45,46]. Due to the defects in the negative selection, the involuted thymus induces elevated T-cell output with self-reactivity, contributing to autoimmune susceptibility and inflammaging, and potentially inducing self-tissue damage [47]. These impairments may dramatically impact one’s predisposition to the SARS-CoV-2 infection and the severity of COVID-19 in elderly people.

Moreover, in [48] increased levels of polyclonal thymic regulatory T-cells (tTreg) were reported, which may intensify the age-related accumulation of Treg cells in the periphery [49,50]. The consequence of such elevations is likely to be a disruption of immune homeostasis or/and imbalanced responses against foreign and self-antigens. It was suggested that the impacts of the alterations associated with both aged bone marrow und thymic involution have a potential influence on the clinical course and severity of COVID-19 in elderly patients [5,46]. One can speculate that dysfunctional lymphopoiesis, characterized by decreased output of lymphocytes, may contribute to lymphopenia, which is known as one of the most important indicators of COVID-19 severity. The decreased number of available lymphocytes could dictate the B-cell mediated humoral immune response at a site of infection. Together with the disturbed T-cell mediated response and autoimmunity—due to the deficient thymopoiesis—this may allow for an unimpeded viral entry and replication and induce self-tissue damage and inflammation.

## 3. How Age-Related Changes in Innate and Adaptive Immunity Induce Inflammaging and May Influence Susceptibility to SARS-CoV-2 and Severity of COVID-19

### 3.1. Age-Related Changes in Innate Immunity and Their Consequences for COVID-19

**Figure 3:**
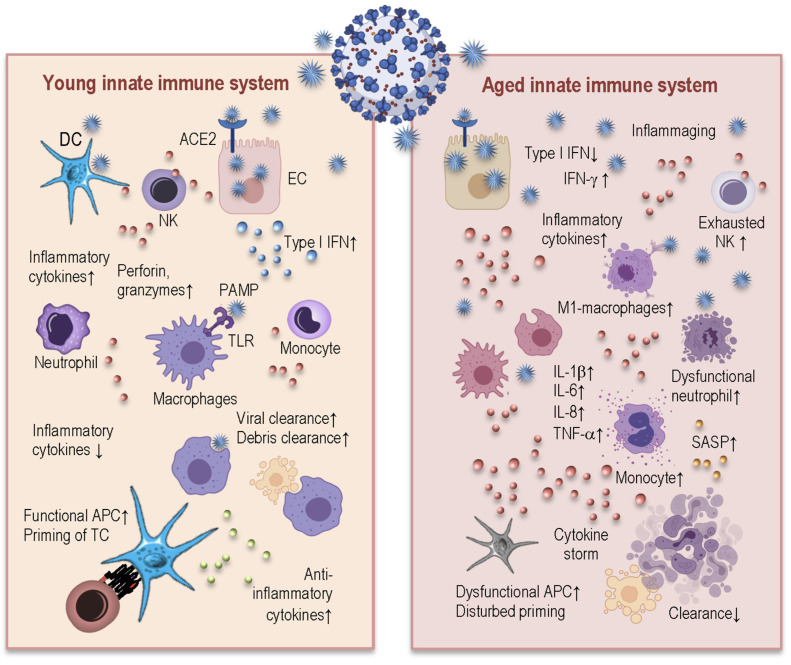
Age-related changes in the innate immune system and their impacts on SARS-CoV-2 infection. The SARS-CoV-2 virus enters endothelial cells by binding to the ACE2 receptor. Once in the host cell (**left**), the virus can be detected by DC, monocytes, and macrophages, inducing local inflammatory and antiviral type I IFN responses that inhibit viral replication and activate and recruit immune cells. The functional APCs of young individuals prime an adaptive immune response, NK cells kill infected cells, and neutrophils clear the cell debris. The innate cells of elderly people (**right**) show impaired functions and the inability to launch a robust type I IFN-response to control virus replication. The inflammaging and SASP attract further dysfunctional inflammatory monocytes, macrophages, and neutrophils to the sites of infection, contributing to the increased local production of cytokines, or “cytokine storm,” and establish an inflammatory feedback loop. These detrimental conditions disturb effective T-cell priming and prevent efficient debris clearance. DC: dendritic cell; NK: natural killer cell; EC: endothelial cell; ACE2: angiotensin-converting enzyme 2; IFN: interferon; PAMP: pathogen-associated molecular pattern; TLR: toll-like receptor; IL: interleukin; TCR: T-cell receptor; SASP: senescence-associated secretory phenotype; APC: antigen-presenting cell; TNF: tumor necrosis factor.

The innate immune system in young and healthy people possesses the exclusive ability to respond immediately to viral infections in a generic way via the initiation of a local inflammatory response for the recruitment and activation of the immune cells, and for the direct elimination of the virally infected cells (Figure 3, left). Additionally, innate immunity plays a central role in the priming of adaptive immune responses by warranting the appropriate activation of T-cells [8].

Immunosenescence influences the proportions and functional ability of innate immune cells, predisposing aged individuals to an inappropriate immune response to a SARS-CoV-2 infection (Figure 3, right). Neutrophils from aged people demonstrate disturbed migration and increased degranulation in the sense that they are not able to efficiently eliminate the pathogen, but retain the ability to secrete highly inflammatory and harmful molecules in their environments. Even more—such elevated levels of proinflammatory molecules cause prolonged survival of the neutrophils via deprivation of apoptosis (87). In fact, the neutrophilia in a patient’s peripheral circulation is recognized as one of the important indicators for poor clinical outcomes with COVID-19. Moreover, the persistent presence of increased levels of neutrophils and monocytes in the blood of COVID-19 patients is associated with the severity of the disease [51,52,53].

The monocytes from elderly patients express the senescent cell marker p21 and show enhanced activation of different pathways linked to oxidative stress, TLR signaling, and NF-κB. Thus, the presence of these aged cells with inflammatory features and/or senescent cells with a senescence-associated secretory phenotype (SASP) may represent one of the mechanisms for exuberant inflammation during a COVID-19 infection [51,54]. Immunosenescence is indeed characterized by the accumulation and persistence of such senescent immune cells with an abnormal secretory phenotype, which produce excessive amounts of inflammatory cytokines, are resistant to apoptosis, and contribute to inflammaging. The cells with SASP are characterized by the production of pro-inflammatory cytokines, chemokines, growth factors, fibronectin, and matrix metalloproteinases, which negatively impact their microenvironment and cause further damage to other cells. Remarkably, the age-related increase in the level of senescent cells is accompanied by a decreased ability of the immune system to remove such senescent cells. This results in further enrichment of the surrounding environment with pro-inflammatory mediators and reactive oxygen species (ROS) and predisposes further neighboring cells to enter immunosenescence [55]. Thus, SASP mediators, together with preexisting upregulation of cytokine expression due to inflammaging, may elicit and also support the excessive hyper-inflammatory conditions in elderly people

Some existing data suggest that the functional exhaustion of peripheral NK cells, similarly to what is observed for CD8^+^ T-cells, contributes to a compromised innate immune response in patients with severe COVID-19 [56]. Multiple studies have reported reductions in NK cells in terms of function and number in COVID-19 patients, which have been associated with reduced clearance of infected and activated cells, and unrestricted elevation of tissue-damaging inflammation factors [57,58].

Innate immune cells have, in general, an exceptionally important function, by serving as antigen-presenting cells (APC) during T-cell priming. To do this properly, innate immune cells should be able to present antigen via the major histocompatibility complex (MHC) together with co-stimulatory receptors during the T-cell/APC interactions. Defects in either of these requirements can have detrimental consequences for the induction of an effective adaptive immune response, and thus negatively modify the disease outcome. It was demonstrated that such essential APCs as monocytes and dendritic cells (DC), obtained from the peripheral blood of patients with acute COVID-19, showed reduced antigen-presenting capacities. When stimulated in vitro, DCs from patients with COVID-19 displayed minimal expression levels of human leukocyte antigen (HLA)-DR, CD80, and CD86 [59]. The patients with severe COVID-19 had reduced expression of HLA-DR on monocytes as well, compared to patients with mild COVID-19 [60,61]. It is well known that immunosenescence also negatively influences antigen presentation. Patients displayed reduced expression levels of CD40, CD86, and MHC class II molecules on DC and monocytes [62]. Additionally, there is an age-related shift in monocyte subsets—a larger non-classical monocyte population [63]. Thus, immunosenescence combined with inflammaging predispose innate immune cells of elderly individuals to dysregulation in their main functions of antigen presentation and T-cell priming. A SARS-CoV-2 infection is apparently capable of further negative modulation of the aged innate immune system towards the inappropriate activation of the adaptive immune system, making aged people particularly susceptible to worse disease outcomes [51].

### 3.2. Age-Related Alterations in Adaptive Immunity and COVID-19

**Figure 4:**
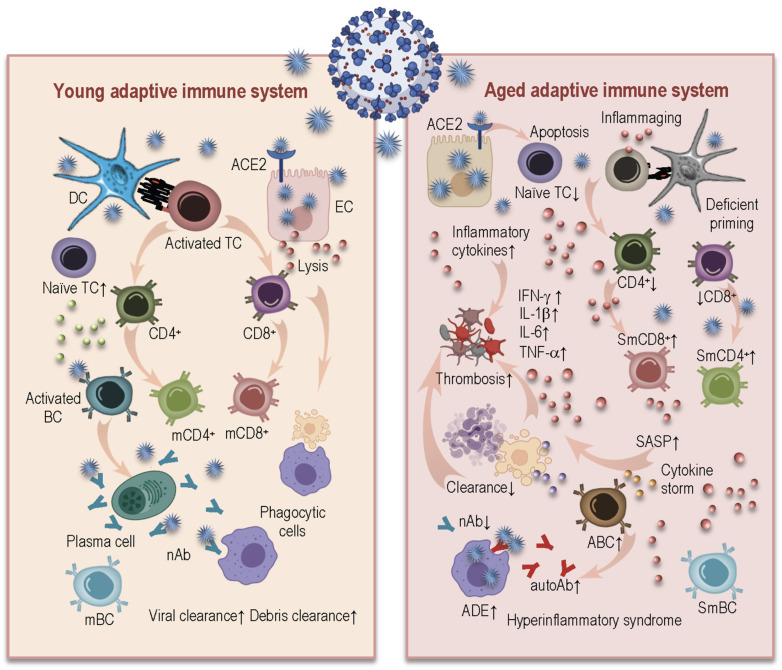
Age-related changes in the adaptive immune system and their impacts on SARS-CoV-2 infection. The SARS-CoV-2 virus can be recognized by dendritic cells of the young immune system (**left**), which release cytokines and present antigens to naïve T-cells for their priming and activation. CD4^+^ T-cells release cytokines and activate B-cells and plasma cells for the production of nAb. Cytotoxic CD8^+^ T-cells are able to directly kill infected cells, preventing the spread of the virus. Neutrophils migrate to the sites of infection to clear infected cell debris. This immune response is capable of resolving the infection and establishing the immune memory. Aged cells of the adaptive immune system (**right**) have multiple deficits which prevent effective antiviral immunity. Decreased numbers of naïve T-cells, limited repertoires of receptors, accumulations of senescent late-differentiated T-cells with decreased proliferative ability, and elevated levels of pro-inflammatory cytokines contribute to the inefficient immune response to the virus. Additionally, severe COVID-19 is characterized by reduced numbers of CD4^+^ and CD8^+^ T-cells. Age-associated B-cells may produce inflammatory cytokines, in particular, TNF-α, affecting the generation of mature B-cells. The reduction in nAb and the elevated levels of non-neutralizing antibodies and autoAb may worsen SARS-CoV-2 infections through ADE, further exacerbating organ damage. SASP mediators, inflammaging, and autoimmunity promote a pro-thrombotic environment, thereby contributing to the hyperinflammatory response observed in severe COVID-19. DC: dendritic cell; EC: endothelial cell; ACE2: angiotensin-converting enzyme 2; TC: T-cell; CD: cluster of differentiation; BC: B-cell; mCD4+: memory helper cell; mCD8+: memory cytotoxic T-cell; mBC: memory B-cell: nAb: neutralizing antibody; IFN: interferon; TNF: tumor necrosis factor; IL: interleukin; SASP: senescence-associated secretory phenotype; SmCD8+: senescent memory CD8+ T-cell; SmCD4+: senescent memory CD4+ T-cell; SmBC: senescent memory B-cell; ABC: age-associated B-cells; ADE: antibody-dependent enhancement.

The outcome of a SARS-CoV-2 infection commonly seen in young, healthy individuals, is the induction of an effective and specific immune response, including both the developing of anti-viral antibodies and the generation of appropriate numbers of viral-specific cytotoxic lymphocytes in order to eliminate the virus and to achieve stable clinical recovery (Figure 4, left).

Aged cells of the adaptive immune system have multiple deficits which prevent effective antiviral immunity (Figure 4, right). The restricted T-cell diversity that was mentioned before represents the major defect limiting their ability to effectively respond to such a novel pathogen as SARS-CoV-2 [64]. Additionally, there is an accumulation of CD28 T-cells, which are not able to conceive essential secondary T-cell activation signaling [46,65,66]. They express multiple senescent markers, such as PD-1 [2] and p16(INK4a) [67], that may inhibit the antiviral T-cell response. The loss of CD28, downregulation of CD27, upregulation of CD57 and KLRG-1, telomere shortening, and impaired secretion of granzyme B combined with the expression of the SASP, are the main hallmarks of senescent T-cells [65,66,68,69,70]. These senescent T-cells with a SASP phenotype promote in an autocrine fashion the recruitment of inflammatory innate cells, which, as mentioned before, are inefficient in their function to remove the senescent cells, contributing additionally to the establishment of inflammaging [46].

Severe COVID-19 is characterized by reduced numbers of CD4^+^ and CD8^+^ T-cells, and peripheral lymphopenia is often associated with functionally exhausted T-cells, having decreased proliferative ability and elevated levels of pro-inflammatory cytokines. In addition to the deficient lympho- and thymopoiesis in the elderly [45], further potential reasons for lymphopenia in severe COVID-19 patients could be related to the relocation of T-cells to airway and lung compartments [71], but also to direct SARS-CoV-2 infections in T-cells [72]. It is also possible that SARS-CoV-2 spike proteins may interact with T-cells through CD26 surface molecules and directly induce apoptosis of T lymphocytes [73], contributing to impairments in T-cell mediated immunity.

Age-related deficits in T-cell functions also influence the humoral response to the virus, as CD4^+^ T-cells provide help via the cytokine production for triggering B-cells to differentiate into immunoglobin-producing plasma cells [74]. The presence of the specific CD4^+^ T-cells in COVID-19 patients has been shown to be decisive for the induction of potent B-cell responses. The levels of spike-specific T-cells were found to correlate with serum titers of IgG and IgA [75]. Convalesced patients displayed robust immune responses with the production of the spike-specific neutralizing antibodies, circulating memory B-cells, and follicular helper T-cells [76].

In general, an age-associated shift from the naïve B-cell phenotype toward memory B-cells was reported [74]. The reduced ability to generate naïve B-cells, combined with the gradual accumulation of dysfunctional memory B-cells, induces an age-related reduction in B-cell repertoire diversity, limiting the antigen recognition of and antigen-specific responses to novel pathogens, such as SARS-CoV-2. Due to alterations in the key stimulatory factors mediating the B-cell response, the generation of long-lived plasma cells may also decrease the response to antiviral vaccines [77].

The main target for neutralizing antibodies that prevent viral attachment to the receptor ACE2 are antibodies that are specific for the spike protein and its receptor-binding domain (RBD) [78]. However, it is still not known how long the antibodies to SARS-CoV-2 will persist. The main consensus is that, despite the fact that neutralizing antibodies may eventually not be detectable after a certain period of time, it is important to have in mind that memory B and T-cells are maintained, making them particularly significant for long-term protection [79]. Due to the age-related functional impairments in the memory cell pools of T and B lymphocytes, long-term protection still remains a critical problem with elderly people.

The formation of long-lived immunity to SARS-CoV-2 was addressed by Kaneko et al., who found that a severe SARS-CoV-2 infection blunted the germinal center (GC) response, which seems to be responsible for the induction of long-lived antibody responses. The absence of GCs was accompanied by a lack of follicular helper T-cells, which are crucial for the generation of GCs. The authors suggested that, with COVID-19, excessive production of TNF-α hinders the establishment of GC responses due to the inhibition of follicular T-cell differentiation and the induction of Th1 responses [80]. Combined with additionally increased levels of TNF-α at baseline, which is typical for inflammaging, this could contribute to the observed phenotype [81]. Other hallmarks of aging, such as a decreased expression of the CD40 ligand on CD4^+^ T-cells, can also affect GC formation, due to the fact that this co-stimulatory molecule plays a critical role in effective T and B-cell interactions [33].

Age-related changes in the immunoglobulin class-switching recombination and somatic hypermutation may also negatively affect the generation of high-affinity antibodies and germinal center formation, which are crucial for the establishment of protective and long-lasting immune responses [82,83] to viral infections. Additionally, a population of age-associated B-cells (ABC) has been identified that produce inflammatory cytokines, in particular, TNF-α, affecting the generation of mature B-cells [84,85]. This population of ABCs possess a unique transcriptional signature and cell surface phenotype, relying for their development and activation on TLR7 and/or TLR9 signaling and the presence of Th1 cytokines [85]. These cells have been found to be linked to autoimmune diseases, and also to COVID-19 [86,87], whereby critically ill patients showed an expansion of the ABC population [51,87]. In addition, non-neutralizing or cross-reactive antibodies produced by B-cells may worsen SARS-CoV-2 infections through antibody-dependent enhancement (ADE), further intensifying tissue damage [88].

Therefore, aged individuals have immune cells with dysregulated immune functions that may be responsible not only for the inefficient annihilation of the virus, but also for the induction of the overwhelming pathological inflammatory responses. As mentioned, this destructive overreaction occurs in part due to chronic low-grade inflammation, age-related high basal TLR activation, modifications in oxidative stress pathways, inflammasome activation, the SASP and elevated senescent cell load, elevated DNA damage, and reduced autophagy [89]. A highly inflammatory environment, SASP mediators, and a senescence-related predisposition to autoimmunity may also initiate the pro-thrombotic pathways in elderly individuals, and so contribute to further indorsing of thrombosis and inflammation in a feed-forward loop, which additionally contributes to the hyperinflammatory, pathological immune response. Thus, a SARS-CoV-2 infection in combination with immunosenescence and inflammaging, may induce an exaggerated inflammatory immune response, promoting a prothrombotic environment and deteriorating the outcome of COVID-19 in aged individuals.

## 4. How CMV May Contribute to Hyperinflammatory Syndrome in COVID-19 Patients

CMV is a very common, worldwide virus that rarely causes disease in healthy, immunocompetent individuals. After millions of years of coevolution, CMV has been efficiently adapted to the human immune system, which is unable to eliminate a CMV infection and the latency that accompanies it, and instead employs a vigorous immune response in maintenance of a homeostatic balance. CMV encompasses a broad host cell spectrum and has the ability to infect different cell types, such as endothelial and epithelial cells, smooth muscle cells, fibroblasts, leukocytes, and dendritic cells. The latent infection is typically established in monocytes and CD34+ cells [90].

As soon as CMV enters host cells, the innate immune system recognizes the virions and triggers several pathways and mechanisms, such as the release of inflammatory cytokines and type I IFN, and the upregulation of surface molecules to reduce the viral replication and to successively prime a high-quality adaptive immune response. This adaptive immune response to CMV is one of the strongest that has ever been documented in humans, comprising both humoral and cellular immunity, and is necessary to control primary CMV infection, after which CMV enters a latency phase. Establishing durable and long-lasting adaptive immunity is essential for the maintenance of CMV latency and for the prevention of lytic infection in healthy individuals [90,91].

The magnitude of the cellular immune response to CMV may not only drive premature immunosenescence; the high frequency of cytolytic T-cells present in infected individuals may be the causative agent and thus exacerbate vascular pathologies. Uncontrolled CMV replication and dissemination may cause life-threatening symptoms and organ damage, which are readily apparent after immunosuppression, clearly demonstrating in this way the essential role of immunity in maintaining asymptomatic co-existence with CMV [92,93].

**Figure 5:**
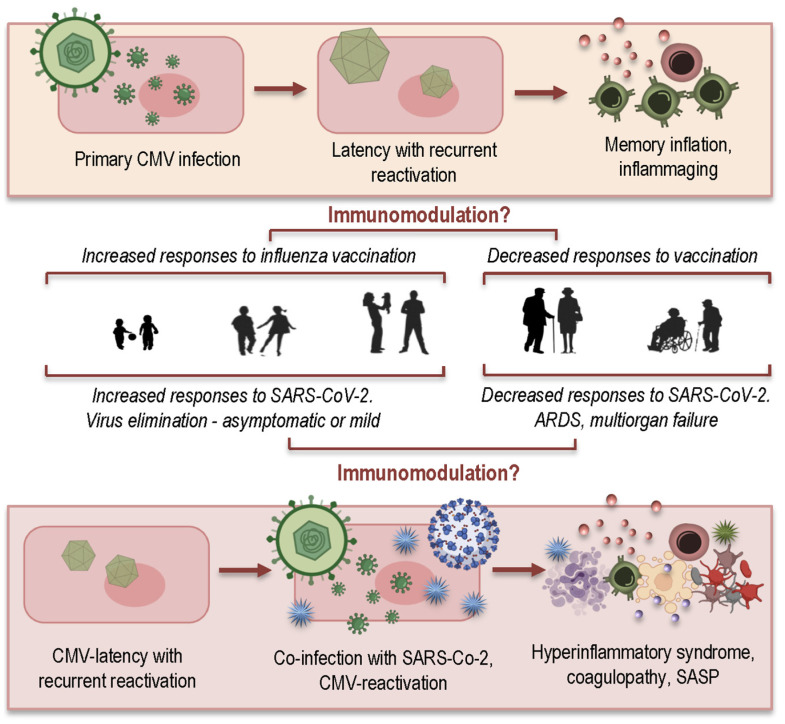
The age-related modulatory effect of a latent CMV infection in response to influenza vaccination, and the hypothetical age-related influences of CMV on the impact of SARS-CoV-2 infections. SASP: senescence-associated secretory phenotype; ARDS: acute respiratory distress syndrome.

Twenty years ago, CMV was recognized as a key player in the immune risk profile (IRP), which was correlated with increased mortality of elderly people within 2 years [94]. CMV exerts a significant influence on the aging immune system [15,16,90,95] and is considered as a driving factor of immunosenescence and inflammaging [96,97]. The primary, mostly asymptomatic infection with CMV (Figure 5, top) is followed by lifelong, latent persistence of the virus, with possible recurrent episodes of reactivation [98]. Due to this repeated exposure to the pathogen, the immune system invests sustained efforts in controlling reactivations of a dormant virus. The accumulation of CMV-specific late-differentiated effector memory T-cells occurs at a cost to the naïve T-cells [99]. So-called “memory inflation” has dramatic consequences for health in the elderly [90,96,100,101,102,103,104], preventing CMV-positive elderly people from generating an effective adaptive immune response to new infections, such as SARS-CoV-2.

Growing evidence indicates that CMV drives the expansion of late-differentiated T-cells with an inflammatory phenotype, contributing to the “inflammaging” phenomenon that is highly associated with the etiology of numerous age-related diseases and may be involved in the immunopathology of COVID-19. Due to its sophisticated immune evasion strategies, CMV is additionally able to directly exert inhibitory effects on such important immune functions as antigen presentation, and NK-cell, B-cell, and T-cell responses [15,105].

The questions that some scientists have recently addressed [106,107,108] on this matter are: Is it possible that CMV is able to reactivate in COVID-19 patients and to act in a combination with SARS-CoV-2? Will this co-infection contribute to pathogenesis, severity, and high mortality in elderly people? It has been reported that 50 % of 38 patients in ICU care, revealed evidence of CMV reactivation [109]. In a letter to the editor, Niitsu et al. reported that CMV reactivation was found in 30% of immunocompetent patients with acute respiratory distress syndrome (ARDS) and was associated with increased mortality [110]. The authors stated that despite the fact that CMV reactivation is particularly alarming in patients with COVID-19—due to ARDS as a common complication of severe COVID-19—the data on CMV infection in these patients are rather scarce.

How might such reactivation occur? It is known that COVID-19 is characterized by elevated levels of TNF-α and IL-6, which may directly stimulate CMV reactivation. Furthermore, SARS-CoV-2 promotes M1-type macrophage polarization, which may also contribute to the reactivation of latent CMV and cause further inflammation [110,111]. Söderberg-Nauclér [108] suggests that, if reactivated CMV acts as a co-infection with SARS-CoV-2, it may have detrimental consequences for patients. Together with CMV, they could suppress T-cells and NK cells, and activate neutrophils and macrophages “in a cascade of events leading to a point of no return from inflammation; and could then affect endothelial cells and thrombocytes to cause coagulopathy and thrombus formation—precisely as observed in COVID-19 patients.” He also summarized that CMV was related to some diseases which raise the odds of having a severe COVID-19 outcome, and additionally that CMV was associated with thrombotic events, such as deep vein thrombosis, stroke, and myocardial infarction—all of which are known as the central complications of COVID-19. Moreover, it was demonstrated that CMV-infected endothelial cells can activate the microthrombi formation in vitro and trigger platelet adhesion and aggregation [92,108,112,113].

CMV also stimulates the production and release of the same chemokines and cytokines that were found to be elevated in COVID-19 patients and to be involved in the cytokine storm—such as MCP-1, MIP-1α, IP10, IL-1β, IL-2, IL-8, IL-10, IL-17, G-CSF, GM-CSF, and TNF-α [112,114,115]. This implies that both viruses may activate similar immune pathways and are both linked to the modulation and overactivation of the immune system and to coagulopathy, which represent two main features of severe COVID-19 [108].

Another question that has not yet been addressed concerns CMV-positive young adults, for whom apparently co-infection with both viruses does not have such dramatic consequences. In this regard, an emerging consensus has appeared that CMV may act differently, depending on the age status of its host (Figure 5, middle). In younger people, CMV infection may enhance immune function, but have a negative impact on immune functions of aged people [107]. Intriguing results were reported in a study by Furman et al., demonstrating that CMV-seropositive young individuals showed better antibody responses to influenza vaccination, augmented CD8^+^ T-cell responses, and elevated levels of circulating IFN-γ compared to seronegative young adults [116]. The generally diminished responses to vaccination in elderly individuals, commonly seen in other studies, were also observed in this study, irrespective of the CMV serostatus of the elderly adults. A better protectional effect against influenza in CMV-seropositive young mice compared to the CMV-seronegative mice was also demonstrated in a murine model, suggesting that this dual age-related effect appears be ubiquitous in vertebrates.

We can speculate that CMV could exert a similar dual immunomodulatory effect during an additional infection with SARS-CoV-2 (Figure 5, bottom). In CMV-seropositive young adults, the presence of CMV may promote an effective immune response to the new pathogen through the immediate and proper activation of both innate and adaptive immune responses, to efficiently eliminate a new invader and produce stabile immune memory. Coinfection of CMV with SARS-CoV-2 may have a more dramatic effect on elderly people due to immunosenescence and inflammaging, resulting in part from the lifelong investment of immunological resources into maintaining CMV-latency. As described previously, the highly elevated proinflammatory environment may induce the reactivation of CMV, and both viruses may synergize in their pathological effects, leading to overactivation of the immune system, the hyperinflammatory syndrome, ARDS, and multiorgan failure.

## 5. Conclusions

The global pandemic will have serious consequences in all aspects of our life. However, it has also provided us with a wealth of new insights and raised many questions, some of which have been answered with great scientific effort, whereas others are still waiting to be answered in the future. We have learned a great number of new facts, have rearranged the old ones, and have reconceived many of them. If previously the immunological, virologic, and epidemiological topics were almost exclusively part of the scientific discourse, they have suddenly become the subject of the general public. As elderly people have been affected by the pandemic to a particularly great extent, and because it was clear from the beginning that the immune system plays a pivotal role in the pathogenesis of this disease, scientists have committed much effort to understanding, reclassifying, and exploring the multiple interactions and interfaces between age-related changes in the immune system and the novel virus. We have realized in a very vivid and clear way how essential and literally vital it is to understand the mechanisms of immunosenescence and the way that the age-related inflammatory environment in the organism may affect the ability of our immune system to generate an adequate immune response against the virus. We also raised the question of how our old companion, latent cytomegalovirus, might be responding and reacting to the new invader. These insights are essential for the development of therapeutic and prophylactic strategies, which should not only be aimed at combating the virus itself, but also at improving the immune functions that decline with age. Many of our questions remain still unanswered and are the subjects of scientific investigation and research. This continuous process of knowledge acquisition is constantly going on, because, even if the current pandemic comes to an end, we should try everything possible to be able to respond to the next challenges—we should be well prepared and armed with the newly-acquired data and new information.

## Data Availability

Not applicable.
